# Influence of light, temperature, and nutrients on microcystin concentration during a winter cyanobacteria-dominated bloom

**DOI:** 10.1093/plankt/fbaf061

**Published:** 2025-11-13

**Authors:** Isabelle M Andersen, Katherine C Rusche, Maggie E Voyles, Alexandra J Bros, Lesley B Knoll

**Affiliations:** Department of Biology, Miami University, 700 E High St, Oxford, OH, 45056, USA; Department of Biology, Miami University, 700 E High St, Oxford, OH, 45056, USA; Wood College of Osteopathic Medicine, Marian University, 3200 Cold Spring Rd, Indianapolis, IN, 46222, USA; Department of Biology, Miami University, 700 E High St, Oxford, OH, 45056, USA; Department of Ecology, Evolution, and Organismal Biology, Iowa State University, 2200 Osborn Dr, Ames, IA, 50011, USA; Department of Biology, Miami University, 700 E High St, Oxford, OH, 45056, USA; Department of Biology, Miami University, 700 E High St, Oxford, OH, 45056, USA

**Keywords:** cyanobacteria, microcystin, winter, light, temperature, nutrients

## Abstract

Freshwater ecosystems are increasingly at risk of experiencing toxin-producing cyanobacterial blooms during the winter due to anthropogenic nutrient loading and climate change. However, understanding how increased light, temperature and nutrient levels impact cyanotoxin production during the winter is limited, as most research has historically focused on blooms during the summer and fall. We conducted 2 × 2 × 2 incubation experiments in February and March to test the individual and interactive effects of light intensity (50 and 150 μmol m^−2^ s^−1^ PAR), elevated temperature (+3°C), and nitrogen and phosphorus enrichment on microcystin concentrations in a *Planktothrix agardhii*-dominated community sampled from Grand Lake Saint Mary’s, a hypereutrophic Ohio reservoir. Microcystin concentration significantly increased with elevated temperature in both months. In February, low light also promoted higher microcystin concentrations, particularly when combined with elevated temperature and nutrient enrichment. In March, nutrient enrichment had individual and interactive effects with temperature that caused higher microcystin concentrations. These results demonstrate that toxin-producing cyanobacteria are active in winter and that climate-driven changes in environmental conditions can interactively increase total toxin concentrations in the water column, even in the non-growing season.

## INTRODUCTION

Toxin-producing cyanobacterial blooms pose a threat to water quality across the aquatic continuum (Wilhelm *et al.,* 2020). The production of cyanotoxins is often difficult to predict, as it can be influenced by a variety of environmental factors. Previous studies have shown that elevated temperatures can enhance toxin concentrations, likely due to toxin production being coupled with faster growth rates (Walls *et al.,* 2018). Additionally, higher nitrogen (N) concentrations can promote cyanotoxin synthesis due to the toxin compounds being N-rich (Jacquemin *et al.,* 2023). There is also evidence that high light can increase toxins, particularly under elevated N conditions (Chaffin *et al.,* 2018). While the effects of these variables on cyanotoxin production have been well studied throughout the summer and fall, less is known about how these environmental variables influence cyanotoxin concentrations during the winter. Climate change is warming winters and shortening ice cover, increasing early-season light and temperature in the photic zone (Cavaliere *et al.,* 2021; Sharma *et al.,* 2021). Simultaneously, anthropogenic activity continues to increase N and phosphorus (P) inputs into aquatic systems, exacerbating the risk of toxic cyanobacteria blooms (Paerl *et al.,* 2016).

*Planktothrix*, a filamentous cyanobacterium capable of producing the hepatotoxin microcystin, is a common winter bloom-forming genus (Grosbois *et al.,* 2024). In this study, we evaluated how the independent and interactive effects of temperature, light, and nutrient availability influenced total microcystin concentration in a *Planktothrix*-dominated phytoplankton community from Grand Lake Saint Mary’s during the winter. We hypothesized that microcystin concentrations would increase with elevated temperature, high light and nutrient enrichment.

## METHODS

### Study site

Water for this experiment was collected from Grand Lake Saint Mary’s (GLSM), a shallow, hypereutrophic reservoir located in Ohio, USA (40.5214°N, −84.4214°W). GLSM has experienced *Planktothrix agardhii-*dominated blooms for decades and microcystin concentrations ranging from 0.03–78 μg L^−1^ (Newell *et al.,* 2024; Lefler *et al.,* 2025). GLSM’s light intensity and temperature were monitored continuously using miniDOT and miniPAR loggers (PME, USA) at 0.5 m depth.

### Experimental design

We conducted two 2 × 2 × 2 experiments, one in late February and one in early March of 2024. For both experiments, water from GLSM was collected from ~ 0.5 m and a subset was used for initial total nitrogen (TN), total phosphorus (TP), microcystin, and phytoplankton community measurements. The remaining water was screened through 63 μm mesh to remove zooplankton and distributed into 1-L flasks. The flasks were exposed, in duplicate, to one of eight treatment combinations consisting of two light treatments, two temperature treatments, and two nutrient treatments. Light treatments included a day-night cycle of low light (LL; ~ 50 μmol m^−2^ s^−1^ PAR) or high light (HL; ~ 150 μmol m^−2^ s^−1^ PAR). Temperature treatments included ambient or elevated (+3°C) conditions to simulate winter warming. Nutrient treatments consisted of ambient or nutrient-enriched (+N&P) conditions that simulated typical storm-driven runoff in Ohio reservoirs (Kelly *et al.,* 2019) with additions of 2.583 μM PO_4_, 203.427 μM NO_3_ and 10.707 μM NH_4_ (N:P = 83 molar). Flasks were stoppered with foam plugs that permitted gas exchange while preventing particulate matter from entering or exiting. After incubating for 8 days in light and temperature-controlled climate chambers, total microcystin samples were collected. Additional laboratory and statistical analyses are described in the Supplemental Material.

## RESULTS

### February

**Fig. 1:**
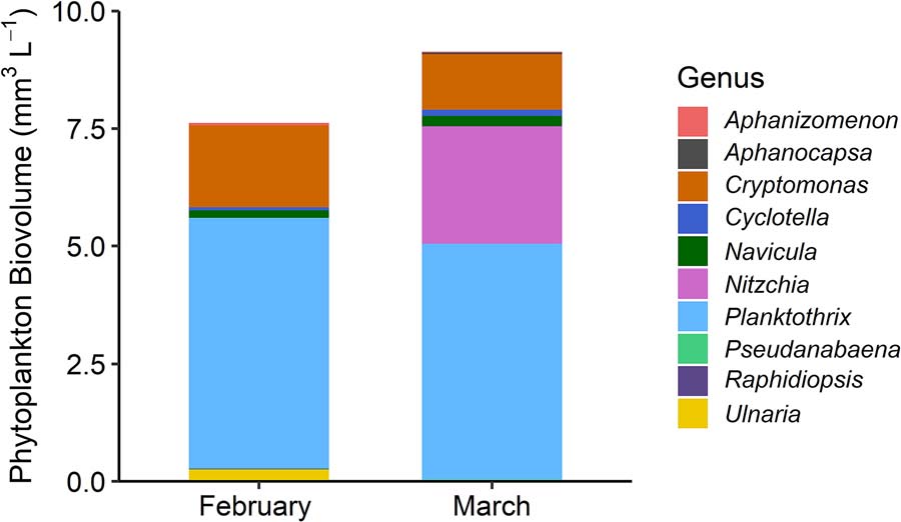
Initial phytoplankton community composition and total biovolume (mm^3^ L^−1^) for February and March. Bars show the total phytoplankton biovolume, with the different colors indicating the relative contribution of each genus.

**Fig. 2:**
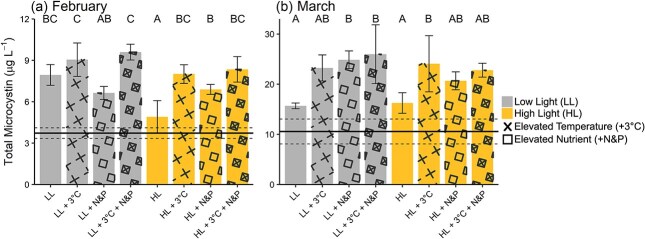
Total microcystin concentrations (μg L^−1^) in response to light, temperature and nutrient treatments for the February (a) and March (b) experiments. Bars represent treatment means with error bars indicating standard deviation. Light treatments included low light (LL) and high light (HL); elevated temperature (+3°C, X pattern) and nutrient enrichment (+N&P, square pattern). Horizontal solid and dotted lines show the mean and standard deviation for initial total microcystin concentrations. Letters above bars indicate statistically significant differences between treatment means (Tables S2 and S4).

Initial TN and TP were 133.69 and 2.97 μM, respectively, resulting in a TN:TP of 45 (molar). Water temperature was 7°C and daytime light intensity averaged 77 ± 88 μmol m^−2^ s^−1^ PAR (mean ± SD). *Planktothrix* comprised 70% of the phytoplankton community (Fig. 1). Initial microcystin concentrations were 3.72 ± 0.38 μg L^−1^ (Fig. 2(a)). Light had a significant effect with LL generally resulting in a higher microcystin concentration (F_1,24_ = 19.03, *P* < 0.001; Table S1). Elevated temperature also had a positive effect on microcystin concentrations (F_1,24_ = 55.15, *P* < 0.001). There was a significant interaction between HL and + N&P that increased microcystin (F_1,24_ = 7.07, *P* < 0.001). Lastly, there was a significant three-way interaction between light, temperature and nutrients (F_1,24_ = 9.10, *P* < 0.001).

### March

Initial TN and TP were 172.13 and 5.91 μM, respectively, resulting in a TN:TP of 29 (molar). Water temperature was 9°C and daytime light intensity averaged 40 ± 49 μmol m^−2^ s^−1^ PAR. *Planktothrix* comprised 55% of the phytoplankton community (Fig. 1). Microcystin concentrations were higher overall with initial concentrations at 10.56 ± 2.48 μg L^−1^ (Fig. 2(b)). Elevated temperature (F_1,24_ = 16.32, *P* < 0.001; Table S3) and + N&P (F_1,24_ = 10.69, *P* < 0.001) had significant individual effects, and interactive effects (F_1,24_ = 6.97, *P* < 0.001) that increased microcystin concentration.

## DISCUSSION

In both February and March, our findings support our hypothesis that elevated temperature increases total microcystin concentrations. Although we did not directly measure growth rate, higher temperatures are known to enhance cyanobacterial growth which is positively correlated with microcystin synthesis (Wagner *et al.,* 2019). Elevated temperature has also been shown to upregulate the microcystin biosynthesis gene *mcyB* (Scherer *et al.,* 2017).

Our March experiment supported our hypothesis that nutrient enrichment increases total microcystin concentrations and our February experiment did not support our hypothesis that high light has a similar effect. Variation in these treatment effects between months may be explained by differences in baseline environmental conditions. TN and TP were higher in March, but the TN:TP was lower. Prior studies show that both high TN and high TN:TP favor cyanotoxin production (Wagner *et al.,* 2019). The high N:P additions likely had a greater impact in March, when ambient TN was lower relative to TP, whereas February’s higher TN:TP may have lessened the individual effect of nutrient enrichment. However, we observed interactions between light and nutrient enrichment and the three-way interaction, indicating that the effect of nutrients depended on both light and temperature. Furthermore, in February, low light significantly increased microcystin concentration. *Planktothrix* prefers low light and can experience photoinhibition under high light, reducing growth and microcystin production (Oberhaus *et al.,* 2007). Ambient light was higher in February, therefore, assuming *Planktothrix* was primarily contributing to total microcystin concentration in this study, exposure to the low light treatment may have reduced light stress, enhancing growth and microcystin production. In contrast, March’s ambient light was already low (likely due to bloom-induced turbidity), so further reduction may not have caused a physiological benefit. However, experimentation exploring the effects of light and nutrient acclimation on potential toxin-producing cyanobacteria growth and microcystin production is required to test these ideas. Overall, these differences between months highlight the importance of background conditions when interpreting treatment effects.

A limitation of this study is the assumption that *Planktothrix* was the primary microcystin-producing genus throughout the experiment. This assumption is based on initial phytoplankton community measurements and previous studies documenting winter *Planktothrix* blooms and elevated total microcystin concentrations in Grand Lake Saint Mary’s (Newell *et al.,* 2024). Although *Aphanocapsa* and *Pseudanabaena* were present at low initial abundances, it is unlikely that these genera contributed to microcystin concentrations, as the microcystin-producing species of these genera are not commonly found in this region (Moura *et al.,* 2018; Cegłowska *et al.,* 2022). It is also possible that other microcystin-producing taxa, not observed in the initial samples, increased in abundance during the incubation and contributed to the final microcystin concentrations. Therefore, without post-incubation microscopy data, we cannot directly link final microcystin concentrations to *Planktothrix* biomass or rule out contributions from other microcystin-producing cyanobacterial taxa as the community could have changed during the incubation period.

## CONCLUSIONS

Our experiment demonstrates that toxin-producing cyanobacteria blooms can persist during winter, a period traditionally considered dormant. Cyanotoxin concentration can be influenced by light intensity, temperature and nutrient availability individually and interactively, and understanding how these environmental factors can influence cyanotoxin concentrations during winter cyanobacteria blooms will help inform management strategies to improve water quality.

## DATA ARCHIVING

The data that support the findings of this study are openly available in *Figshare at*  https://doi.org/10.6084/m9.figshare.29627465

## Supplementary Material

GLSM_toxins_supp_fbaf061
