# Supplementary material for: Influence of light, temperature, and nutrients on microcystin concentration during a winter cyanobacteria-dominated bloom
Source: J Plankton Res. 2025 Nov 13;47(6):fbaf061. doi: 10.1093/plankt/fbaf061 (PMC12613827; doi:10.1093/plankt/fbaf061)
Supplement: GLSM_toxins_supp_fbaf061 [file glsm_toxins_supp_fbaf061.docx]

**Title**: Influence of Light, Temperature, and Nutrients on Microcystin Production During a Winter *Planktothrix* Bloom

**Authors**: Isabelle M. Andersen 0000-0002-8674-6238, Katherine C. Rusche 0009-0004-4467-1807, Maggie E. Voyles 0009-0001-0044-5232, Alexandra J. Bros 0009-0008-4423-4358, Lesley B. Knoll 0000-0003-0347-5979

**Supplemental Materials**

*Study Site and Sampling Details*

GLSM has a surface area of 52 km^2^, an average depth of ~1.5 m. This reservoir has a 238 km^2^ watershed that consists mostly of crop and livestock agricultural land use, resulting in high levels of nonpoint source run-off (Newell et al., 2024).

Samples for the experiment were gathered on February 22, and March 14, 2024. A subset of the initial water samples was acidified below 2 pH, and stored at 4°C for later analysis of initial total nitrogen (TN) and total phosphorus (TP) concentrations. Additional subsamples were frozen at 4°C for later determination of initial total microcystin concentrations. To determine GLSM’s phytoplankton community composition at time of sampling, water was also preserved with Lugol’s iodine for microscopic identification and enumeration.

*Laboratory Analysis*

Following three freeze-thaw cycles, cyanotoxin samples were analyzed using an ABRAXIS Microcystins-ADDA (OH) Enzyme-Linked Immunosorbent Assay (ELISA) Microtiter Plate (Gold Standard Diagnostics, Horsham, PA, USA) to measure total microcystin concentrations. The ELISA kits were run in accordance with the US Environmental Protection Agency “Method 546” (Ohio EPA, 2015). A Spectra iMax plate reader was used to analyze the plate results.

Phytoplankton samples were settled using Utermöhl chambers and analyzed using an inverted microscope at 400x (Zeiss model 473307-9902). For each sample, twenty independent fields of view were examined and phytoplankton observed were counted and identified to genus level (over 400 natural units per sample counted). Dimensions corresponding to the genera’s respective geometric shape (Hillebrand et al., 1999) were measured using an ocular micrometer. Biovolume (mm^3^ L^-1^) for each sample was calculated by multiplying the genera’s volume by the number of units counted per mL.

Total nutrient samples were persulfate digested prior to analysis with a Lachat QC 8000 FIA autoanalyzer (Lachat Instruments, Loveland, CO, USA).

*Statistical Analysis*

To assess the main and interactive effects of light, temperature, and nutrient supply on total microcystin concentrations, we conducted a three-way analysis of variance (ANOVA) using the aov() function. The *emmeans* package was used to estimate means and conduct pairwise comparisons between all treatment combinations using Tukey’s Honest Significant Difference. All analyses were done using R (Version 4.5.1, R Core Team 2025).

**Tables**

**Table S1.** Results of a three-way ANOVA evaluating the individual and interactive effects of light, temperature, and nutrient enrichment on total microcystin concentrations for the February experiment.

| Treatment Effect | df | Sum sq | Mean sq | F value | *p*-value |
| --- | --- | --- | --- | --- | --- |
| Light | 1 | 12.86169 | 12.86169 | 19.02916 | <0.001 |
| Temp | 1 | 37.27239 | 37.27239 | 55.14534 | <0.001 |
| Nutrient | 1 | 1.237353 | 1.237353 | 1.830691 | 0.188655 |
| Light:Temperature | 1 | 0.116377 | 0.116377 | 0.172182 | 0.681866 |
| Light:Nutrient | 1 | 4.779913 | 4.779913 | 7.071989 | 0.013726 |
| Temperture:Nutrient | 1 | 0.020278 | 0.020278 | 0.030002 | 0.863937 |
| Light:Temperature:Nutrient | 1 | 6.148369 | 6.148369 | 9.096651 | 0.005973 |

**Table S2.** Pairwise contrasts from the February three-way ANOVA showing differences in total microcystin concentrations between treatment combinations of high light (HL), low light (LL), elevated temperature (3°C), and nutrient enrichment (N&P).

| Contrast | Mean Estimate | Standard Error | df | t value | *p*-value |
| --- | --- | --- | --- | --- | --- |
| HL - LL | -3.04 | 0.58 | 24 | -5.23 | <0.001 |
| HL - HL+ 3°C | -3.11 | 0.58 | 24 | -5.34 | <0.001 |
| HL - LL+ 3°C | -4.15 | 0.58 | 24 | -7.14 | <0.001 |
| HL - HL + N&P | -1.99 | 0.58 | 24 | -3.43 | 0.039 |
| HL - LL + N&P | -1.73 | 0.58 | 24 | -2.98 | 0.101 |
| HL - HL + 3°C + N&P | -3.45 | 0.58 | 24 | -5.93 | <0.001 |
| HL - LL + 3°C + N&P | -4.70 | 0.58 | 24 | -8.08 | <0.001 |
| LL - HL+ 3°C | -0.07 | 0.58 | 24 | -0.12 | 1 |
| LL - LL+ 3°C | -1.11 | 0.58 | 24 | -1.91 | 0.557 |
| LL - HL + N&P | 1.05 | 0.58 | 24 | 1.79 | 0.627 |
| LL - LL + N&P | 1.31 | 0.58 | 24 | 2.25 | 0.361 |
| LL - HL + 3°C + N&P | -0.41 | 0.58 | 24 | -0.70 | 0.996 |
| LL - LL + 3°C + N&P | -1.66 | 0.58 | 24 | -2.85 | 0.128 |
| HL+ 3°C - LL+ 3°C | -1.04 | 0.58 | 24 | -1.79 | 0.629 |
| HL+ 3°C - HL + N&P | 1.11 | 0.58 | 24 | 1.91 | 0.555 |
| HL+ 3°C - LL + N&P | 1.37 | 0.58 | 24 | 2.36 | 0.303 |
| HL+ 3°C - HL + 3°C + N&P | -0.34 | 0.58 | 24 | -0.58 | 0.999 |
| HL+ 3°C - LL + 3°C + N&P | -1.59 | 0.58 | 24 | -2.74 | 0.159 |
| LL+ 3°C - HL + N&P | 2.16 | 0.58 | 24 | 3.71 | 0.021 |
| LL+ 3°C - LL + N&P | 2.42 | 0.58 | 24 | 4.16 | 0.007 |
| LL+ 3°C - HL + 3°C + N&P | 0.70 | 0.58 | 24 | 1.21 | 0.921 |
| LL+ 3°C - LL + 3°C + N&P | -0.55 | 0.58 | 24 | -0.94 | 0.978 |
| HL + N&P - LL + N&P | 0.26 | 0.58 | 24 | 0.45 | 0.999 |
| HL + N&P - HL + 3°C + N&P | -1.45 | 0.58 | 24 | -2.49 | 0.243 |
| HL + N&P - LL + 3°C + N&P | -2.70 | 0.58 | 24 | -4.65 | 0.002 |
| LL + N&P - HL + 3°C + N&P | -1.71 | 0.58 | 24 | -2.95 | 0.106 |
| LL + N&P - LL + 3°C + N&P | -2.96 | 0.58 | 24 | -5.10 | <0.001 |
| HL + 3°C + N&P - LL + 3°C + N&P | -1.25 | 0.58 | 24 | -2.15 | 0.413 |

**Table S3.** Results of a three-way ANOVA evaluating the individual and interactive effects of light, temperature, and nutrient enrichment on total microcystin concentrations for the March experiment.

| Treatment Effect | df | Sum sq | Mean sq | F value | *p*-value |
| --- | --- | --- | --- | --- | --- |
| Light | 1 | 17.88321 | 17.88321 | 1.68271 | 0.206897 |
| Temp | 1 | 173.4635 | 173.4635 | 16.32194 | 0.000476 |
| Nutrient | 1 | 113.5979 | 113.5979 | 10.68893 | 0.003245 |
| Light:Temp | 1 | 0.810126 | 0.810126 | 0.076228 | 0.784839 |
| Light:Nutrient | 1 | 38.65623 | 38.65623 | 3.637334 | 0.068536 |
| Temp:Nutrient | 1 | 74.1017 | 74.1017 | 6.972553 | 0.014325 |
| Light:Temp:Nutrient | 1 | 0.272902 | 0.272902 | 0.025679 | 0.874029 |

**Table S4.** Pairwise contrasts from the March three-way ANOVA showing differences in total microcystin concentrations between treatment combinations of high light (HL), low light (LL), elevated temperature (3°C), and nutrient enrichment (N&P).

| Contrast | Mean Estimate | Standard Error | df | t value | *p*-value |
| --- | --- | --- | --- | --- | --- |
| HL - LL | 0.57 | 2.31 | 24 | 0.25 | 1.000 |
| HL - HL+ 3°C | -7.83 | 2.31 | 24 | -3.40 | 0.041 |
| HL - LL+ 3°C | -7.00 | 2.31 | 24 | -3.04 | 0.089 |
| HL - HL + N&P | -4.43 | 2.31 | 24 | -1.92 | 0.551 |
| HL - LL + N&P | -8.63 | 2.31 | 24 | -3.74 | 0.019 |
| HL - HL + 3°C + N&P | -6.54 | 2.31 | 24 | -2.84 | 0.132 |
| HL - LL + 3°C + N&P | -9.74 | 2.31 | 24 | -4.22 | 0.006 |
| LL - HL+ 3°C | -8.40 | 2.31 | 24 | -3.65 | 0.024 |
| LL - LL+ 3°C | -7.57 | 2.31 | 24 | -3.28 | 0.053 |
| LL - HL + N&P | -5.00 | 2.31 | 24 | -2.17 | 0.404 |
| LL - LL + N&P | -9.19 | 2.31 | 24 | -3.99 | 0.011 |
| LL - HL + 3°C + N&P | -7.11 | 2.31 | 24 | -3.09 | 0.080 |
| LL - LL + 3°C + N&P | -10.30 | 2.31 | 24 | -4.47 | 0.003 |
| HL+ 3°C - LL+ 3°C | 0.84 | 2.31 | 24 | 0.36 | 1.000 |
| HL+ 3°C - HL + N&P | 3.40 | 2.31 | 24 | 1.48 | 0.812 |
| HL+ 3°C - LL + N&P | -0.79 | 2.31 | 24 | -0.34 | 1.000 |
| HL+ 3°C - HL + 3°C + N&P | 1.29 | 2.31 | 24 | 0.56 | 0.999 |
| HL+ 3°C - LL + 3°C + N&P | -1.90 | 2.31 | 24 | -0.82 | 0.990 |
| LL+ 3°C - HL + N&P | 2.57 | 2.31 | 24 | 1.11 | 0.947 |
| LL+ 3°C - LL + N&P | -1.63 | 2.31 | 24 | -0.71 | 0.996 |
| LL+ 3°C - HL + 3°C + N&P | 0.45 | 2.31 | 24 | 0.20 | 1.000 |
| LL+ 3°C - LL + 3°C + N&P | -2.74 | 2.31 | 24 | -1.19 | 0.928 |
| HL + N&P - LL + N&P | -4.20 | 2.31 | 24 | -1.82 | 0.613 |
| HL + N&P - HL + 3°C + N&P | -2.12 | 2.31 | 24 | -0.92 | 0.981 |
| HL + N&P - LL + 3°C + N&P | -5.31 | 2.31 | 24 | -2.30 | 0.333 |
| LL + N&P - HL + 3°C + N&P | 2.08 | 2.31 | 24 | 0.90 | 0.983 |
| LL + N&P - LL + 3°C + N&P | -1.11 | 2.31 | 24 | -0.48 | 1.000 |
| HL + 3°C + N&P - LL + 3°C + N&P | -3.19 | 2.31 | 24 | -1.38 | 0.856 |

**References**

Newell, S. E., Doll, J. C., Jutte, M. C., Davidson, J. L., McCarthy, M. J. and Jacquemin, S. J. (2024) Drivers and mechanisms of harmful algal blooms across hydrologic extremes in hypereutrophic grand lake st marys (Ohio). *Harmful Algae*, 138, 102684.

R Core Team. 2025. *R: a language and environment for statistical computing*. Vienna, Austria: R Foundation for Statistical Computing.
